# Neurosarcoidosis With Panhypopituitarism: Two Cases and Literature Review

**DOI:** 10.1210/jcemcr/luae141

**Published:** 2024-08-06

**Authors:** Ewelina Niedzialkowska, Tatjana Blazin, Daniel Shelden, Eric D Buras

**Affiliations:** Department of Internal Medicine, Corewell Health William Beaumont University Hospital, Royal Oak, MI 48073, USA; Department of Internal Medicine, Corewell Health William Beaumont University Hospital, Royal Oak, MI 48073, USA; Division of Endocrinology, Diabetes and Metabolism, Department of Internal Medicine, Corewell Health William Beaumont University Hospital, Royal Oak, MI 48073, USA; Division of Metabolism, Endocrinology and Diabetes (MEND), Department of Internal Medicine, University of Michigan, Ann Arbor, MI 48105, USA

**Keywords:** neurosarcoidosis, hypopituitarism, panhypopituitarism, arginine vasopressin deficiency

## Abstract

Neurosarcoidosis (NS) with hypothalamic-pituitary (HP) involvement (HP-NS) is a rare clinical condition, conferring variable hormonal deficits that are typically irreversible. Here, we present 2 cases of NS with panhypopituitarism. The first patient presented with cauda equina syndrome and arginine vasopressin deficiency, while the second developed recurrent optic neuritis and vision loss in the setting of a sellar mass. In the first case, neurological symptoms resolved after therapy with high-dose glucocorticoids, infliximab, and methotrexate; while in the second, visual restoration followed resection of the granulomatous tissue and immunosuppressive therapy. In both cases, pituitary dysfunction persisted despite neurological improvement. We contextualized the presentations and outcomes through a literature review of HP-NS case reports and case series. This revealed high rates of extraneurologic sarcoidosis in HP-NS patients with panhypopituitarism, while underscoring the need for hormonal replacement—as endocrinopathies rarely respond to sarcoidosis-directed immunosuppression.

## Introduction

Sarcoidosis is a multisystem disease characterized by noncaseating granulomas that affects the central nervous system in 5% to 10% of cases (1). Hypothalamic-pituitary neurosarcoidosis (HP-NS) occurs in 0.5% to 2.5% of sarcoidosis patients, accounts for 1% of sellar masses, and carries a mortality rate approaching 10% (2-4). Endocrinopathies may herald HP-NS disease presentation and involve both anterior and posterior pituitary hormone deficits. Based on available reports, hypogonadism—present in 87.5% to 88.8% of HP-NS cases—appears to the most common hormonal deficiency affecting the anterior pituitary (3, 4). This is followed in prevalence by thyroid dysfunction (56%-67.4%), hyperprolactinemia (48.4%-55%), adrenocorticotropin (ACTH) deficiency (37%-48.4%), and growth hormone (GH) deficiency (30%-50%) (3, 4). Arginine vasopressin deficiency (AVP-D) has also been observed in 50% to 58% of patients with HP-NS (4). Panhypopituitarism—defined as complete loss of anterior and posterior pituitary function—has been described in 13 published cases (3). Here, we discuss 2 cases of NS manifesting as panhypopituitarism, both of which initially presented with neurological dysfunction: The first with cauda equina syndrome—a rare manifestation, occurring in only 6.5% of individuals with NS (5)—and the second with an invasive sellar mass and optic neuritis. In each circumstance, endocrinopathies required sustained hormone replacement therapy.

## Case Presentation

### Case 1

A 32-year-old man presented with bilateral lower extremity pain, polyuria, polydipsia, and fecal-urinary incontinence. He had previously been diagnosed with NS at another institution 9 months prior to admission. At that time, magnetic resonance imaging (MRI) of the brain showed diffuse leptomeningeal enhancement, while computed tomography of the chest showed tree-in-bud opacities with reactive hilar lymphadenopathy. On lumbar puncture, cerebrospinal fluid analysis revealed elevated protein and angiotensin-converting enzyme levels, as well as leukocytosis with lymphocytic predominance. Hilar lymph node biopsy was consistent with noncaseating granuloma. After this tissue-based NS diagnosis, the patient started prednisone and methotrexate but did not adhere to the regimen. On admission to our hospital, physical examination revealed gait ataxia, gynecomastia, urinary incontinence, and positive Romberg sign.

### Case 2

A 61-year-old woman presented to the emergency department complaining of a 2- to 3-week history of bilateral vision loss more severe on the left. She had a history of recurrent optic neuritis (3 episodes, starting 2 years prior) with imaging suggestive of pituitary macroadenoma at the time of its initial diagnosis (convex superior margin and fullness of the pituitary gland).

## Diagnostic Assessment

### Case 1

**Figure 1.**
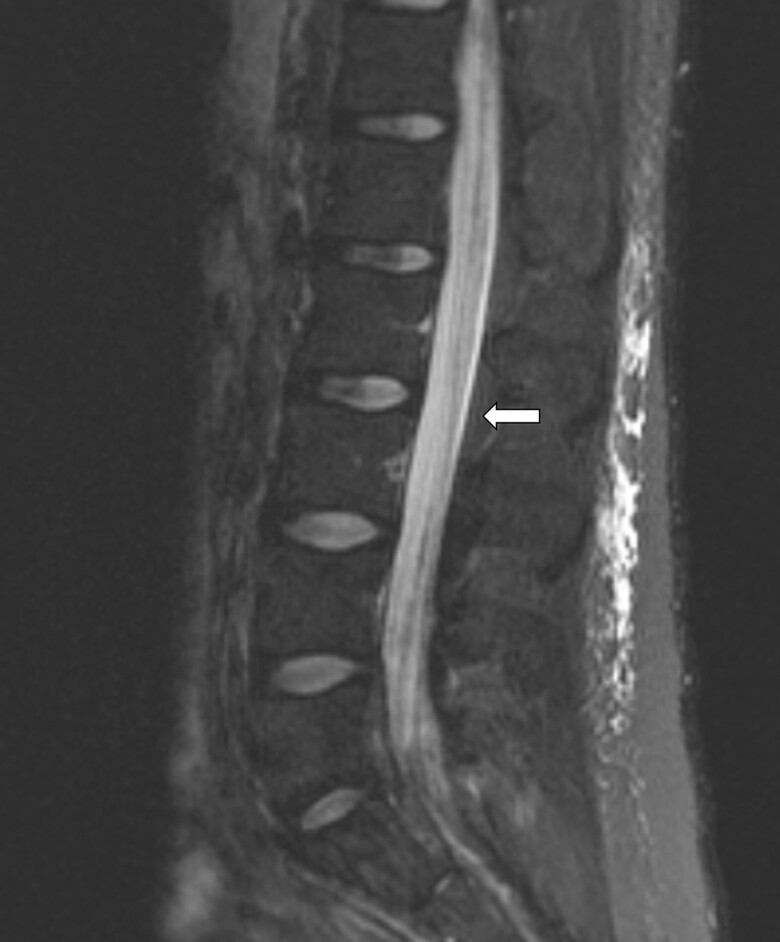
Extensive nodular enhancement involving the leptomeninges of the lumbar spine (arrow) as well as lumbar cauda equina nerve roots involvement.

**Figure 2.**
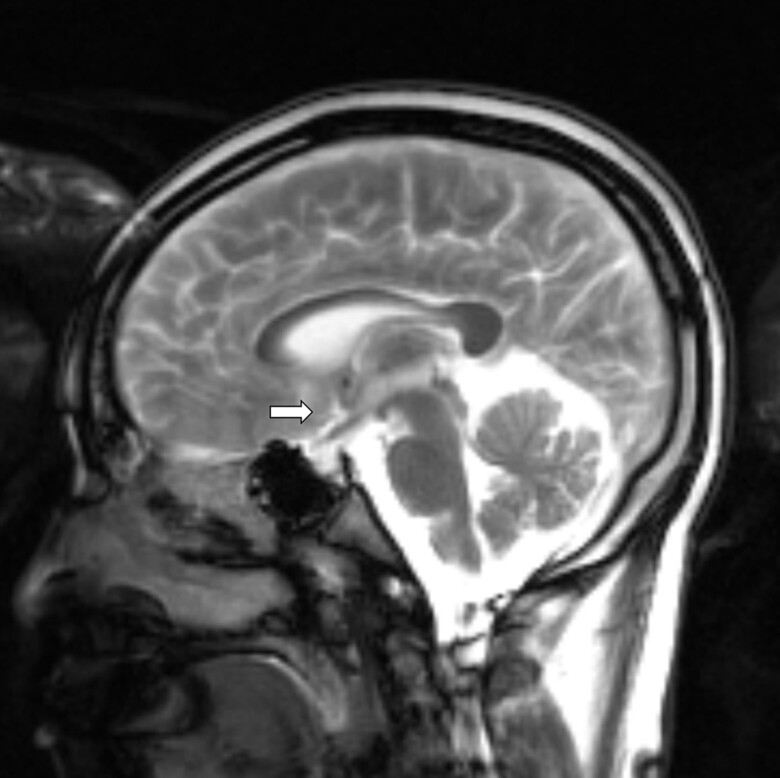
Brain magnetic resonance imaging with numerous small nodular enhancement involving leptomeninges and cerebrospinal fluid space supratentorially and infratentorially, pituitary stalk (arrow), interpeduncular cistern, and periaqueductal perivascular space.

Spinal MRI demonstrated extensive nodular enhancement of the cervical and thoracic leptomeninges, as well as the lumbar cauda equina nerve roots (Fig. 1). Brain MRI showed numerous small nodular enhancements involving the supratentorial and infratentorial leptomeninges and cerebrospinal fluid space; as well as the pituitary stalk, interpeduncular cistern, and periaqueductal perivascular space (Fig. 2). Laboratory evaluation revealed urine specific gravity 1.001, luteinizing hormone (LH) 0.4 mIU/mL (N 0.6-1.2 mIU/mL), follicle-stimulating hormone (FSH) 1.1 mIU/mL (N 1-12 mIU/mL), ACTH <5 pg/mL (<1.1 pmol/L) (N 6-46 pg/mL; 1.32-10.12 pmol/L), cortisol 2.1 µg/dL (57.9 nmol/L) (N 2.9-19.4 µg/dL; 80-535 nmol/L), prolactin 10.5 µg/L(10.5 µg/L) (N 2-18 µg/L), total testosterone 4 ng/dL (0.13 nmol/L) (N 240-1080 ng/dL: 8.32-37.45 nmol/L), free testosterone 0.1 ng/dL (0.0035 nmol/L) (N 4.8-19 ng/dL; 0.16-0.65 nmol/L), sex hormone–binding globulin 36 nmol/L (N 11-78 nmol/L), thyrotropin 0.58 mIU/L(N 0.4-4.5 mIU/L), free 3,5,3′-triiodothyronine 1.5 pg/mL (2.3 pmol/L (N 1.7-3.7 pg/mL; 2.6-5.7 pmol/L), free thyroxine (free T4) 0.6 ng/dL (7.72 pmol/L) (N 0.7-1.5 ng/dL; 9-19 pmol/L), insulin-like growth factor-1 (IGF-1) 52 ng/mL (6.7 nmol/L) (N 71-234 ng/mL; 9.28-30.6 nmol/L), copeptin pro-AVP <2.8 pmol/L (N < 13.2 pmol/L), urine osmolality 72 mOsmol/kg, serum osmolality 297 mOsmol/kg (N 275-295 mOsmol/kg).

### Case 2

**Figure 3.**
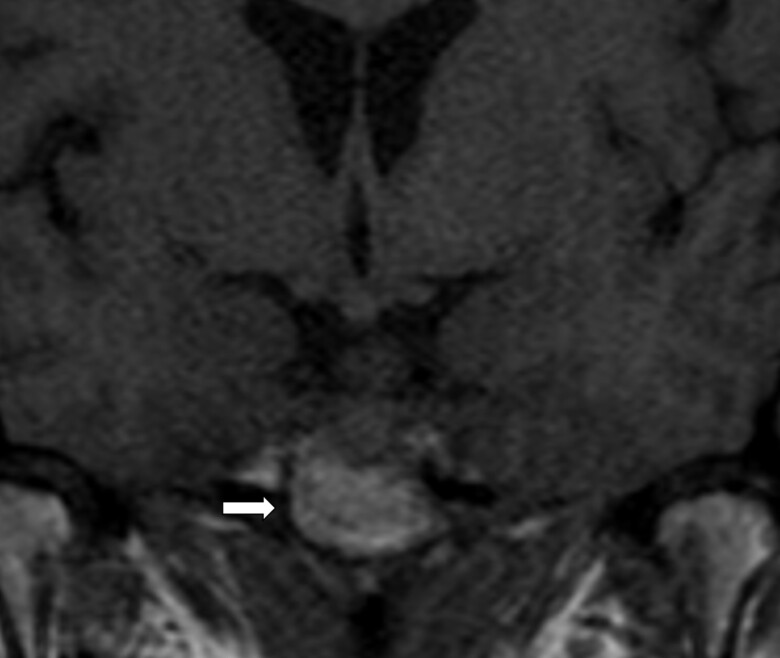
Magnetic resonance imaging of the brain: 1.8 cm sellar mass (arrow) causing deviation of the optic chiasm.

Brain MRI identified a 1.8-cm sellar mass (Fig. 3) causing deviation of the optic chiasm. This exhibited traumatic invasion of bilateral cavernous sinuses and spread along the left tentorium. Hormonal evaluation showed morning cortisol of 0.395 µg/dL (10.9 nmol/L) (N 2.9-19.4 µg/dL; 80-535 nmol/L) (with subsequent abnormal ACTH stimulation test—maximum cortisol level 0.29 µg/dL; 7.9 nmol/L), FSH 1.8 mIU/mL (postmenopausal >10 years, N 19-100 mIU/mL), LH < 0.1 mIU/mL (N 14.2-52 IU/mL), IGF-1 < 25 ng/mL (<3 nmol/L) (N 71-234 ng/mL; 9.28-30.6 nmol/L), free T4 0.27 ng/mL (3.46 pmol/L) (N 0.7-1.5 ng/dL; 9-19 pmol/L), thyrotropin 1.29 mIU/L (0.4-2.5 mIU/L), and prolactin 3 µg/L (N 2-10 µg/L).

## Treatment

### Case 1

To control the sarcoidosis flare, the patient started pulse-dose methylprednisolone followed by prednisone 60 mg daily (subsequently tapered to 20 mg daily), as well as methotrexate 20 mg weekly and infliximab infusions. Fecal-urinary incontinence resolved with immunosuppression; however, high urinary volume persisted. Levothyroxine was started at 100 mcg daily for hypothyroidism; and intranasal desmopressin acetate (DDAVP) enabled improvement of polyuria.

### Case 2

The patient started stress-dose glucocorticoids perioperatively and underwent posterior ethmoidectomy and posterior septectomy. A tan, very fibrous mass, not grossly consistent with meningioma or adenoma, was identified and resected. Pathological assessment of the 1.6 × 1.1 × 0.2-cm sample identified granulomatous inflammation, allowing diagnosis of sarcoidosis in the absence of identifiable infectious organisms. After surgery the patient started levothyroxine 125 mcg daily; and glucocorticoids were tapered before discharge to achieve hydrocortisone doses of 15 mg in the morning and 10 mg in the afternoon. After a brief course of mycophenolate mofetil, the patient began a methotrexate-based sarcoidosis regimen enabling resolution of neurological symptoms. Postoperatively, she also developed persistent polyuria (3-5 L daily) for which a low-dose DDAVP (25 mcg orally daily) was initiated and uptitrated to 150 mcg daily with improvement in symptoms. At follow-up visits, she was found to have normal-range free T4 and undetectable FSH and LH.

## Outcome and Follow-up

### Case 1

Nine months after initial presentation, MRI demonstrated that nodules within the brain and leptomeningeal enhancement of the cervical and lumbar spine had largely resolved. The patient continued levothyroxine replacement, while maintenance DDAVP was required to quell persistent polyuria. Free and total testosterone levels remained low; and the patient sought sperm preservation through the urology department prior to initiating testosterone replacement.

### Case 2

Nine months after granuloma resection and initiation of immunosuppression, the patient achieved resolution of visual abnormalities. She continued methotrexate 15 mg weekly for long-term management of sarcoidosis. She self-discontinued DDAVP but was able to adequately hydrate to counteract polyuria. Multiple serum and urine osmolality measurements indicated persistent, mild AVP-D; and she transiently restarted DDAVP before again self-discontinuing. Bone densitometry of the lumbar spine and femoral neck were within normal limits, while laboratory testing ruled out hypercholesterolemia and hyperglycemia. Despite persistently low IGF-1, GH supplementation was not pursued.

## Discussion

We evaluated 2 patients with NS and panhypopituitarism. Despite successful surgical and immunosuppressive management of sarcoidosis with markedly improved neurological function, both patients required hormone replacement therapy months following their initial presentations.

To better contextualize these cases, we performed a literature review analyzing the incidence, demographics, and clinical presentation of patients with NS and combined anterior-posterior pituitary dysfunction. We reviewed 105 articles, excluded 90 due to missing laboratory data, language other than English, and alternate diagnosis. The remaining 15 articles (10 case reports, 5 case series) described 41 total cases (Table 1). Fifty-nine percent of the patients evaluated were male, and mean age at presentation was 37 years. Gonadal insufficiency was the most frequent endocrinopathy (85.4%), followed by hypothyroidism (73%), adrenal insufficiency (51.2%), and GH deficiency (39%). Hypoprolactinemia occurred in 43.9% of patients, while hyperprolactinemia was seen in 4.8%. Eleven patients (26.8%) presented with panhypopituitarism. Among these, 54% were male, and mean presentation was at 40 years. Frequencies of individual pituitary axis abnormalities broadly correspond with those reported in a previous multicenter study that included 24 HP-NS patients with either anterior or posterior hormone deficiencies (3). In that analysis, and in our review, prevalence of GH deficiency is potentially falsely low, as several included case descriptions basing the diagnosis only on IGF-1 levels (which may underestimate GH deficiency in older individuals).

**Table 1.** Demographics, hormonal dysfunctions, extraneurologic involvement, and treatment of patients with anterior and posterior pituitary dysfunction

Publication	Patient No.	Age, y	Sex	Thyroid axis	Adrenal axis	Gonadal axis	Growth hormone axis	Prolactin	ADH	Extraneurologic involvement	Treatment
Sukuraman, 2019 (6)	1	15	F	↓	↓	↓	↔	↔	↓	Cervical and auricular lymph nodes	GC, LTX, OCT, DDAVP
Waard et al, 2017 (7)	1	40	M	↔		↓			↓	Pulmonary	GC, DDAVP
Anthony et al, 2016 (4)	3	59	M	↓	↓	↓			↓	Pulmonary	GC, MMF, TT, LTX, DDAVP
	4	48	M	↔	↓	↓	↓	↓	↓	Pulmonary	AZT, GC, DDAVP, TT
O’Relly et al, 2015 (8)	1	22	M	↓	↓	↓			↓	Cervical lymph nodes	DDAVP, GC, INFX, LTX, TT
Tanaka et al, 2012 (9)	1	58	F	↔	↔	↓	↓	↔	↓	Subclavian lymph nodes	GC, DDAVP
Motta et al, 2008 (10)	1	41	F	↓	↔		↔	↑	↓	paratracheal, retrocaval and paraaortic lymph nodes	GC, LTX, CFM, DDAVP, radiation
**Miyoshi et al, 2007** (**11**)	**1**	**77**	**M**	**↓**	**↓**	**↓**	**↓**	**↔**	**↓**	**Pulmonary**	**GC, LTX, DDAVP**
Randeva et al, 2002 (12)	1	57	F	↓	↓	↓		↑	↓		GC, AZT, DDAVP, LTX
Sweeney et al, 1995 (13)	1	51	F	↓		↓			↓	Pulmonary	
Hidaka et al, 1987 (14)	1	21	M	↓		↓	↓		↓	Pulmonary, uveitis	GC, LTX, HCG
Langrand et al, 2012 (3)	**3**	**30**	**F**	**↓**	**↓**	**↓**	**↓**	**↓**	**↓**	**Sinus, skin, bone, liver**	**GC**
	7	37	M	↔	↔	↓	↓	↔	↓		GC, MTX, MMF
	8	32	M	↓	↓	↓	↔	↓	↓	Pulmonary, skin, gonads	GC, CPM, MMF, AZT
	**9**	**23**	**M**	**↓**	**↓**	**↓**	**↓**	**↔**	**↓**	**Pulmonary**	**GC, MTX, CPM, INFX**
	11	55	M	↓	↓	↓	↔	↔	↓	Pulmonary, ophthalmic, sinus	GC
	12	31	M	↔	↔		↔	↔	↓	Ophthalmic	GC
	14	56	F	↔	↔	↔	↔	↔	↓	Pulmonary	GC, MTX
	15	41	F	↓	↔	↓	↔	↓	↓	—	GC
	16	42	F	↓	↔	↓	↔	↓	↓	Pulmonary, ophthalmic, bone, liver	GC, MTX
	17	31	F	↓	↔	↓	↔	↓	↓	Pulmonary, sinus	GC, MTX
	**19**	**32**	**M**	**↓**	**↓**	**↓**	**↓**	**↓**	**↓**	**Pulmonary, ophthalmic, sinus**	**GC, MMF**
	**22**	**53**	**M**	**↓**	**↓**	**↓**	**↓**	**↔**	**↓**	**Pulmonary, ophthalmic**	—
Bihan et al, 2007 (15)	**1**	**44**	**F**	**↓**	**↓**	**↓**	**↓**	**↔**	**↓**	**Pulmonary, sinus**	**GC**
	2	23	M	↓	↓	↔	↓	↔	↓	Pulmonary, sinus, GI	GC, MTX
	**3**	**32**	**M**	**↓**	**↓**	**↓**	**↓**	**↓**	**↓**	**Pulmonary, skin, sinus**	**GC**
	**4**	**47**	**F**	**↓**	**↓**	**↓**	**↓**	**↓**	**↓**	**Pulmonary, sinus, ophthalmic**	**GC**
	5	27	M	↓		↓		↔	↓	Pulmonary, sinus, ophthalmic	GC
	**8**	**19**	**F**	**↓**	**↓**	**↓**	**↓**	**↔**	**↓**	**Pulmonary, sinus, joints**	**GC, MTX, CPM, HCQ**
	9	38	M		↓	↓	↔	↔	↓	Pulmonary, sinus, cardiac	GC, MTX
Tabuena et al, 2004 (16)	1	27	M	↓	↔	↓		↓	↓	Pulmonary	GC
	2	32	F	↔				↓	↓	Pulmonary	GC
	3	26	M	↔	↓	↓		↓	↓	Pulmonary	GC
	4	18	M	↓	↓	↓		↓	↓	—	GC
Bullmann et al, 2000 (17)	1	40	F	↔	↔	↔	↔	↓	↓	—	GC
	2	41	F	↔	↔	↓	↔	↓	↓	—	GC
	3	27	M	↓	↔	↓	↔	↓	↓	Pulmonary, muscular	GC, CPM
	4	21	M	↓	↔	↓	↔	↓	↓	Lymph nodes, pulmonary, GI, ophthalmic	GC
	5	26	M	↓	↔	↓	↔		↓	Cardiac, pulmonary	GC
**Niedzialkowska, 2024 (current work)**	**1**	**32**	**M**	**↓**	**↓**	**↓**	**↓**	**↔**	**↓**	**Pulmonary, lymph nodes**	**GC, LTX, DDAVP, MTX, INFX**
	**2**	**54**	**F**	**↓**	**↓**	**↓**	**↓**	**↓**	**↓**	**Ophthalmic**	**GC, LTX, DDAVP, MTX**

Patients with panhypopituitarism are presented in bold.

Abbreviations: ADH, antidiuretic hormone; AZT, azathioprine; CPM, cyclophosphamide; DDAVP, desmopressin acetate; F, female; GC, glucocorticosteroids; GI, gastrointestinal; HCQ, hydroxychloroquine; INFX, infliximab; LTX, levothyroxine; M, male; MMF, mycophenolate mofetil; MTX, methotrexate; OCT, oral contraceptive therapy; TT, testosterone therapy.

From the data presented, we were unable to definitively compare the extent of neurological involvement in NS patients with panhypopituitarism vs those with AVP-D in whom one or more anterior pituitary axes were intact. Regarding extraneurologic disease, pulmonary findings were most common among patients in our data set, affecting 27 of 41 (65.9%) patients. These were followed in frequency by sinus (24%), ocular (21%), lymph node (14%), skin (7%), liver (4.7%), cardiac (4.8%), bone (4.8%), gastrointestinal tract (4.8%), and muscle (2.4%) involvement. In patients with panhypopituitarism, pulmonary (81%), sinus (45%), and ophthalmic (36%) involvement were more frequent—suggesting that, despite the limited data, individuals with complete loss of pituitary function might have greater risk of extraneurologic disease.

Consistent with prior reports (3, 4), all patients in our data set received glucocorticoids. Individual patients received additional immunosuppressive agents, including methotrexate (11/41), mycophenolate mofetil (4/41), azathioprine (3/41), cyclophosphamide (3/41), infliximab (2/41), and hydroxychloroquine (1/41). One patient also underwent radiation therapy. Reported treatment rates for endocrinopathies were surprisingly low in the cases evaluated. Specifically, DDAVP was reportedly administered to 11 of 41 patients with AVP-D (27%), levothyroxine to 9 of 30 patients with hypothyroidism (30%), and testosterone to 3 of 23 male patients with hypogonadism (13%). A single female patient with hypogonadism received oral contraceptive therapy (8.3%). Whether reporting was incomplete or accounted for only deficiencies corrected within the few days following HP-NS diagnosis is unclear.

There is no consensus treatment regimen for NS. While high-dose glucocorticoids are the therapeutic mainstay, these are often coupled with various immunomodulators, consistent with those employed in the cases reviewed. Notably, Anthony et al (4) found no correlation between neurological improvement (defined radiologically) and amelioration of endocrine deficiencies. And, while beneficial effects of glucocorticoids on HP axis recovery have been proposed, restoration of pituitary function is rare (16). For instance, on 4-year follow-up of NS patients who achieved marked neuroimaging improvement following immunosuppression, only 8% recovered any pituitary function (3).

Both patients discussed in this report presented with neurological symptoms—cauda equina syndrome and optic neuritis in cases 1 and 2, respectively. It is noteworthy, however, that symptomatic endocrinopathies may represent the presenting complaint in HP-NS. Indeed, clinically apparent HP dysfunction preceded diagnosis of sarcoidosis in 13 of 24 HP-NS patients described by Langrand et al (3). Both patients described here also had extraneurologic findings on sarcoidosis diagnosis—pulmonary involvement in case 1 and ocular involvement in case 2. This is broadly consistent with the comparatively higher rate of extraneurologic disease in HP-NS patients with panhypopituitarism.

In both cases, patients were treated with glucocorticoid/methorexate regimens that also included infliximab (case 1) or mycophenolate mofetil (case 2). Each patient improved symptomatically, and central nervous system lesions markedly regressed in case 1. These outcomes reflect the previously reported effectiveness of immunosuppression in promoting resolution of neurological symptoms and abnormal neuroimaging in HP-NS (3). The specific effect of combinatorial immunosuppression in cauda equina syndrome was highlighted by Bou et al (18), who demonstrated that while glucocorticoids have a durable effect in only 21.4% of cases, the addition of methotrexate/infliximab combination therapy yields resolution in 75%. Critically, in the cases described here, HP dysfunction did not resolve following immunosuppression. This is consistent with multiple reports (3, 15) indicating that the vast majority of HP-NS endocrinopathies (8%-22%) persist even after resolution of neuroimaging findings. As such, sarcoidosis patients with suspected HP-NS require clear definition of HP axis function, early initiation of hormone replacement therapy, and long-term endocrinology follow-up.

## Learning Points

- NS with HP dysfunction is a rare clinical entity but should be suspected in patients presenting with central endocrinopathies.
- Radiological improvement of NS does not correlate with improvement in hormonal dysfunction. Endocrine recovery is rarely reported; and patients should be started on appropriate replacement therapy.

## Data Availability

Data sharing is not applicable to this article as no data sets were generated or analyzed during the current study.

## Abbreviations

ACTH: adrenocorticotropic hormone
AVP-D: arginine vasopressin deficiency
FSH: follicle-stimulating hormone
GH: growth hormone
HP: hypothalamic-pituitary
IGF-1: insulin-like growth factor-1
INFX: infliximab
LH: luteinizing hormone
MMF: mycophenolate mofetil
MRI: magnetic resonance imaging
MTX: methotrexate
NS: neurosarcoidosis
T4: thyroxine
